# Psychometric properties of the Inventory of Interpersonal Problems (IIP-C) used with a clinical sample of adolescents: a preliminary study

**DOI:** 10.21307/sjcapp-2021-010

**Published:** 2021-04-27

**Authors:** Pravin Israel, Johannes Hendrik Langeveld

**Affiliations:** VID Specialized University Stavanger Norway; Senter for Familieomsorg, Familieomsorg Norway; University of Stavanger, Stavanger, Norway

**Keywords:** Inventory of Interpersonal Problems IIP-C, adolescent psychiatry, psychometric properties, interpersonal problems

## Abstract

**Background:**

Interpersonal problems are consistently identified with psychopathology that often has its onset in adolescence. Most of the commonly used instruments in child and adolescent psychiatry target non-interpersonal problems. The Inventory of Interpersonal Problems (IIP) is a widely studied and utilized instrument in the adult mental health field.

**Aims:**

This study aimed to examine the psychometric properties of the IIP (circumplex version) used with a clinical adolescent population. Method: Sixty-two adolescents (13-17 years) who received treatment in a child and adolescent mental health clinic (CAMHS) were included in the study. To establish reliability and validity, we conducted confirmatory factor analyses, internal consistency, and validity analyses.

**Results:**

Confirmatory analyses did not show optimal model fit. However, other indices like CFI and TLI were promising. The reliability of the eight scales was in the same range as previous studies and acceptable. There were expected significant correlations between IIP-C scales and the broadband scales of Youth Self-report (YSR).

**Conclusion:**

The pioneer nature and its clinical focus are strengths however, there is a need for more research. The promising results are encouraging, and future research could also explore how best to bring the instrument into the digital age.

## Introduction

Interpersonal problems are consistently identified with psychopathology such as major depression, anxiety, alcohol and drug dependence, and maladjusted personality (1–4). These psycho-pathologies have their onset in adolescence (13-18 years) and persist into adulthood (5), suggesting that assessing adolescents’ interpersonal problems is an important agenda for mental health professionals. Many instruments used in Child and Adolescent Mental Health Services (CAMHS) target non-interpersonal problems such as feelings, thoughts, and behaviors. The Inventory of Interpersonal Problems (IIP) is the only self-report instrument that assesses interpersonal problems and is widely used in adult mental health field (6). The aim of this study was to examine the psychometric properties of the IIP when used with adolescents (14-18 years) treated in CAMHS.

### IIP background and adaptations

The IIP was developed in the crucible of clinical work with people seeking psychotherapy. Horowitz et al. (7) noted that people’s interpersonal complaints dominated the content and themes of their therapy. This was different from the symptom focus of assessments and outcome measures. This gave impetus to develop the IIP, which consisted of 127 items and had six scales. The alpha coefficients ranged from .82 to .93, and test-retest reliability ranged between .80 to .90 over a 10-week period. Further, the IIP displayed high sensitivity to therapeutic change, and its usefulness as a clinical tool gained popularity.

Over the years, researchers keen on using the measure created various versions of the IIP to best suit their needs. One line of development was motivated by a need for a clinic-friendly measure that was short and brief (8–10). In a review, Hughes & Barkham (11) reported ten shorter versions of IIP. The IIP short version’s popularity has grown globally and it is available in various languages and versions (12). Additionally, studies using IIP have been reported from Italy (13), Turkey (14), and also international students (Chinese) in USA (15). Cronbach alpha for the short form IIP’s ranged from .68 to .92 (10, 16).

Another line of development was influenced by the research-based traditions of interpersonal relationships and the circumplex model (17, 18). Circumplex models emerge from interpersonal theories and provide a framework to organize interpersonal content along two bipolar dimensions of dominance and love. The connotation of dominance is personal control and agency, while love alludes to affiliation, closeness and friendliness (19). The interpersonal problems are organized in a circular fashion, and each of the eight scales is a blend of varying amount of dominance and love. Horowitz et al. (10) postulated that there was a common complaint factor, a general tendency to report distress that varied from patient to patient. However, removing the distress factors by ipsatizing the items and subjecting the scales to PCA, a two-factor solution emerged, reminiscent of the dominant-submissive and hostile-friendly dimensions derived from interpersonal circumplex tradition. Inspired by this discovery and possibility of multiple uses, Alden et al. (20) constructed circumplex scales for the IIP. This work yielded a measure with 64 items and eight scales that were labelled following the Sullivan and Leary tradition (21, 22): Domineering (PA), Vindictive (BC), Cold (DE), Socially avoidant (FG), Nonassertive (HI), Exploitable (JK), Overly Nurturant (LM), & Intrusive (NO). Alden et al. (20) reported alpha coefficients in the range of .72 -.85 for the eight scales. Other studies using IIP-C have also reported alpha coefficients in the same range (23, 24). Additionally, some studies examined the expression of IIP-C compared to the hypothesized circumplex structure. One study based on a community sample reported a bifactor model with an acceptable fit (CFI= .97; RMSEA= .078) (25). Citing previous studies that conducted a formal test of the model (26–28), Monsen et al. (29) conducted confirmatory factor analysis based on a mixed sample of a clinical (n = 374) and a normal reference sample (n = 355). The baseline result prior to applying modification indexes was not acceptable (TLI = .928; CFI = .933; RMSEA = .114). However, after relaxing constraints on three correlations in the normal sample and seven correlations in the clinical sample, the model achieved acceptable fit to the data (TLI = .946; CFI = .977; RMSEA =.078).

### Measuring interpersonal problems in adolescents

Two sources provide guidelines for what psychometric instruments are recommended for CAMHS in Norway: the Norwegian Directorate of Health, and the Norwegian Association for Child and Adolescent Psychiatry. Guidelines propose assessing a wide range of factors that cover anamnestic information, symptoms, somatic functioning, cognitive abilities, i.e., intelligence, family and social network, and functioning (30). The Youth Self Report - YSR (31) is widely endorsed for use in CAMHS. While the YSR provides comprehensive information, only two scales address interpersonal issues. The narrow-band competency scales assess a combination of pro-social activities and global questions regarding how well the child/youth gets along with family and friends. The syndrome scale called social problems consists of 11 items of which six items directly address problematic interpersonal problems with peers. Other recommended instruments are the Strengths and Difficulties Questionnaire (SDQ; 32) and HoNOSCA (33), which are primarily screening instruments. Some other instruments target adolescents’ relationships, like The Inventory of Parent and Peer Relationship (IPPA; 34), which measures adolescents’ trust, communication and alienation. While these instruments provide information that aims to aid in well-informed diagnostics and treatment choices, none of them offers a comprehensive assessment of the adolescents’ interpersonal problems.

The IIP is clinically relevant and robust across the wide variety of versions, translations and populations. Further, it found sound footing in the research-based traditions of the interpersonal circumplex. The potential of this combination was highlighted by Wiggins: Horowtiz’ IIP “*opened promising new directions of research in such areas as attachment theory, clinical judgement, depression, and psychotherapy*” (21, p. 226). Encouraged by these accolades, we wondered if the IIP was useful also with adolescents. A search of the literature showed IIP being used with adolescents (35–37). However, these studies are few, and to our knowledge, there are no studies that have examined the psychometric properties of the IIP-C in a clinical adolescent population.

### The aims of the study

The primary aim of this study is to assess the psychometric properties of the IIP-C used with a clinical sample of adolescents (14-18 years) treated in CAMHS. We are primarily interested in examining if the IIP-C results from an adolescent population yield the same factor structure as we see in an adult clinical population. To establish validity, we use a commonly used tool in CAMHS - the Youth Self Report (YSR), and its two broadband syndrome scales of internalizing and externalizing problems. We expect to see a correlation pattern where IIP-C dominant scales will be significantly correlated with the externalizing scale. Conversely, IIP-C submissive scales will be significantly correlated with the internalizing scale. Additionally, the YSR scale “social problems” was of particular interest due to its implicit concordance with interpersonal content. We expected social problems to have high correlation with all of the IIP-C scales.

## Method

### Procedures

The study was part of a larger study about parent involvement in usual clinical practice. The regional ethics committee (REK-III), University of Bergen approved the project. All the participants and parents of the participants under 16 years of age consented to participate in the study. All new referrals to the clinics received printed information regarding the research study either before or shortly after the first consultation at the clinic. The primary therapist was present during the assessments and answered questions the adolescents may have had. Since the IIP-C was not used in adolescents before, we first asked a panel of six therapists and another panel of five adolescents who were currently receiving in-patient treatment, to provide feedback regarding the instrument. We were interested in their assessment of the comprehension level, instructions, burden of time to complete the assessments and its level of difficulty. Based on their feedback and recommendation, no changes were made to the Norwegian translation.

### Participants

A total of 62 adolescents between 14-18 years of age participated in the study. The participants were referred to and treated in outpatient clinics in CAMHS. Information regarding referral reason was available for 70% (n = 44) of the clinical sample. Forty percent (n = 18) was referred due to being sad/depressed 16 % (n = 7) for behavioral problems. The others were spread over a variety of referral reasons like anxiety/phobia (2%), school problems (7%), hyperactivity (9%), eating disorders (9%), autistic symptoms (7%) and (10%) were referred for various other reasons. We had only two exclusion criteria - psychosis and clinician evaluated developmental disorder, i.e., IQ < 70. The mean age of the sample was 15.7 years (SD= 1.04), and 69 % were girls. Fifty-six per cent of the adolescents lived with both parents, 32 % had permanent residence with their mothers, and a small group (2.4 %) commuted between the mother and father. The rest (9.6 %) lived in adoptive homes and residential care facilities.

## Measures

### Inventory of Interpersonal Problems (IIP-C)

The IIP-C consists of 64 problem statements assessed in relation to a significant person and indicated on a Likert scale based on agreement (0 = not at all - 4 = extremely). The first 39 items begin with “*It is hard for me to* (for example) *trust other people and keep things private from other people*.” The second 25 items start with “*things I do too much*” (for example),”*I fight with other people too much,”* and “*I am too sensitive to criticism”*. The items form eight scales that are projected on to the interpersonal circumplex. The IIP-C scales have their own abbreviations which are as follows: Domineering/Controlling (PA), Vindictive/Self-centered (BC), Cold/Distant (DE), Socially inhibited (FG), Nonassertive (HI), Exploitable (JK), Overly Nurturant (LM), and Intrusive/Needy (NO). The IIP-C was translated into Norwegian (38) and reported good reliability with alpha coefficients in the range of .67 – .87 (39). This is the version of the translation that we used in the current study.

### The Youth Self Report (YSR)

The YSR questionnaire consists of 112 items that adolescents report on a 3-point Likert scale (0 = Not true, 1 = somewhat or sometimes true, and 3 = very true or often true). There are three scales assessing the competence (Activities, Social, and School), eight narrow-band syndrome scales (Anxious/Depressed; Withdrawn/Depressed; Somatic Complaints; Social Problems; Thought Problems; Attention Problems; Rule-Breaking Behavior and Aggressive Behavior). The two broadband scales are made up of Anxious/Depressed, Withdrawn/Depressed and Somatic Complaints (Internalizing Problems) and Rule-breaking behavior and Aggressive behavior (Externalizing problems). The alpha coefficients for the syndrome scales range from .71 - .95 (40). The Norwegian version was found to have acceptable psychometric properties (41).

## Statistical analyses

Scale scores were computed as the mean of the items in the actual subscale (10). We based our CFA model on the bi-factor model described by Wilson et al. (25). Previous studies of IIP-C showed the presence of a general distress factor and accounting for it produced the well-known circumplex structure. Therefore, instead of using an orthogonal model we directly used the bi-factor model. The eight IIP-C scales were loaded with their theoretical values of -.707, -.5, 0 (twice), .5 and .707 (twice) for the two factors of Dominance and Love, and the general factor represented by a loading of .707 (see Figure 1A). The two latent factors and general distress factor were constrained to be uncorrelated. The standardized coefficients are presented in Figure 1B. We used the statistical package Strata, to conduct confirmatory factor analyses and used following fit indices: the root mean square effort of approximation (RMSEA), comparative fit index (CFI), Tucker Lewis index (TLI), and the standardized mean square residual (SRMR). Reliability analyses included Cronbach’s alpha, inter-item correlation, item-total correlation and intercorrelations of the IIP-C scales. Validity analysis was conducted using bivariate correlations- two-tailed using the statistical package SPSS version 26.

**FIGURE 1A.**
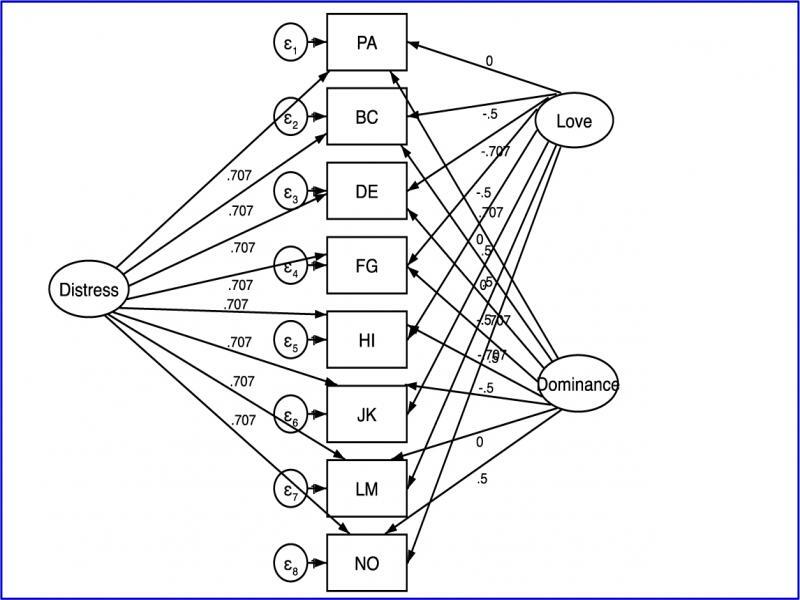
The hypothesized bi-factor model of the IIP-C used with adolescents

**FIGURE 1B.**
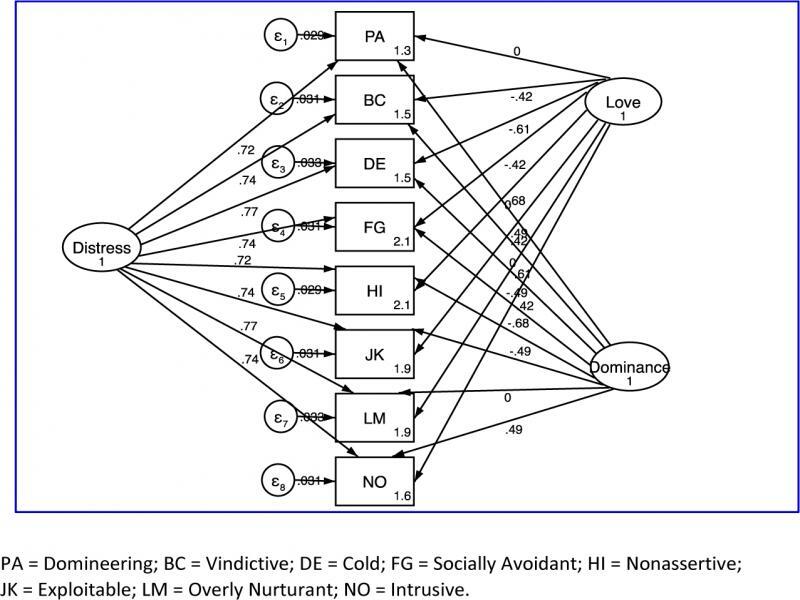
Standardized coefficients of the bi-factor CFA solution with Distress PA = Domineering; BC = Vindictive; DE = Cold; FG = Socially Avoidant; HI = Nonassertive; JK = Exploitable; LM = Overly Nurturant; NO = Intrusive.

**FIGURE 2.**
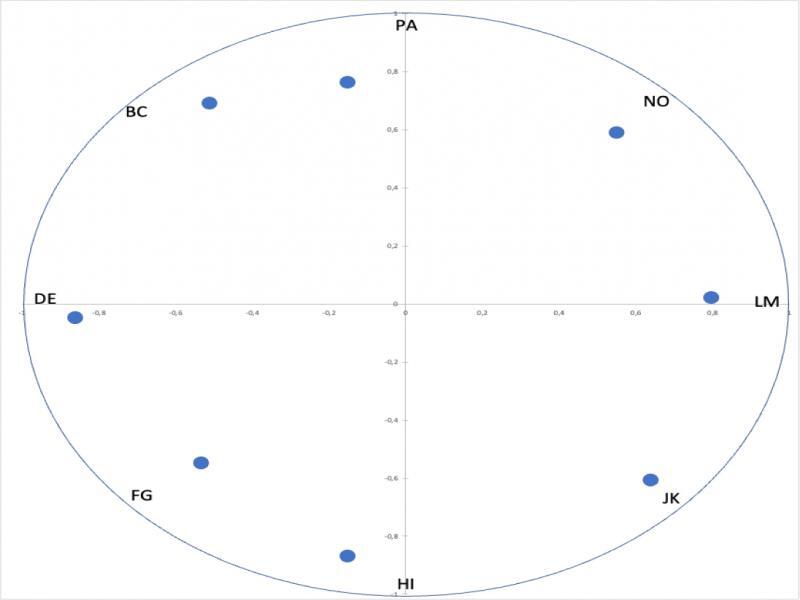
The Circumplex structure of the IIP-C used with adolescents PA = Domineering; BC = Vindictive; DE = Cold; FG = Socially Avoidant; HI = Nonassertive; JK = Exploitable; LM = Overly Nurturant; NO = Intrusive.

## Results

### Confirmatory factor analyses

The fit indices were *χ* ^2^_25_ = 51.073; RMSEA = .131 (.079 - .182); CFI = .918; TLI = .908; SRMR= 0.281. The RMSEA exceeded the acceptable level of .08, implying that the estimated model was significantly different from the theoretical model. However, CFI and TLI were above > .90 and SRMR was below .80, indicating that the model may hold some promise.

### Reliability of the IIP-C scales

Table 1 shows Cronbach’s alpha for the total score (.93) and the eight scales (range = .69 -.84). The highest α coefficient was for HI and lowest for PA. The item-total correlation had a range between .08 and .72. Two items had an item-total correlation below .20. They were item 44, *I am too independent*, and item 29, *it is hard for me to put another's needs before my own*. Deleting these items did not affect the alpha coefficient of the total IIP-C scale. However, removing item 44 from the subscale increased the α coefficient of PA by .04, and removing item 29 increased the α coefficient of BC by .02, which are small-to-medium changes and therefore, the items were retained in subsequent analyses.

**TABLE 1.** Cronbach’s alpha, inter-item correlations and item-total correlations

	Mean (SD)	Inter-item correlation average	Item-total correlation range	Cronbach’s alpha
PA	7.46 (4.86)	0.24	0.09 – 0.53	0.69
BC	8.7 (5.64)	0.26	0.15 – 0.65	0.74
DE	7.98 (5.84)	0.30	0.24 – 0.70	0.78
FG	11.79 (6.97)	0.36	0.28 – 0.71	0.82
HI	12.3 (7.65)	0.40	0.49 – 0.72	0.84
JK	10.87 (6.83)	0.35	0.31 – 0.67	0.81
LM	10.65 (5.49)	0.25	0.23 – 0.43	0.72
NO	8.93 ( 5.86)	0.24	0.24 – 0.55	0.71
Total	78.79 (34.02)	0.30	0.16 – 0.65	0.93

1 *Note*. PA = Domineering; BC = Vindictive; DE =Cold; FG = Socially Avoidant; HI = Nonassertive; JK = Exploitable; LM = Overly Nurturant; NO = Intrusive

The intercorrelations of the IIP-C scales are summed up in table 2 and show the expected patterns of correlations of circular structures. The range of correlations based on raw scores was from -.07 to .66, and the correlations based on ipsative scores ranged from -.62 to .46. As expected from a circumplex structure, there were higher correlations with adjacent scales and lower or negative correlations with scales on the circle's opposite side. The pattern of correlations was more pronounced with scales based on ipsatized scores than the raw scores.

**TABLE 2.** Intercorrelations of IIP-C: above diagonal are raw scores and below are ipsative scores

	PA	BC	DE	FG	HI	JK	LM	NO
PA	1	.661**	.494**	.312*	0.124	-0.072	0.207	0.22
BC	.459**	1	.693**	.412**	.319*	-0.023	0.228	0.184
DE	-0.031	.346**	1	.768**	.691**	.295*	.394**	0.152
FG	-.293*	-0.218	.330**	1	.771**	.498**	.522**	0.137
HI	-.622**	-.464**	0.157	.346**	1	.713**	.622**	0.084
JK	-.503**	-.714**	-.524**	-0.088	.373**	1	.729**	.280*
LM	-0.117	-.356**	-.647**	-.376**	-0.146	.340**	1	.470**
NO	0.156	0.04	-.421**	-.581**	-.616**	-0.057	.277*	1

1 *Note*. N = 62. Correlations ** = *p* > .01 (twotailed); * = *p* > .05

2 PA = Domineering; BC = Vindictive; DE =Cold; FG = Socially Avoidant; HI = Nonassertive; JK = Exploitable; LM = Overly Nurturant; NO = Intrusive

The eight scales were rotated to converge maximally with the theoretical locations to obtain and a visual representation of their location in the interpersonal circumplex. Figure 1 shows a clear circumplex structure and the scales are placed close to their hypothesized positions.

### External validity: correlation with expected broadband syndromes

There was a moderate correlation between the YSR total syndrome scale (M = 56.6, SD = 11.39) and the IIP-C total score (M = 78.69, SD = 34.02), r(62) = 0.59, *p* < 0.00, suggesting a certain degree of overlap between the instruments. Table 3 shows the correlation patterns between the scales of the two instruments. IIP-C scales based on raw scores and ipsative scores were used to minimize the effect of general factor in IIP-C. Not all the correlations were statistically significant. There were higher correlations between IIP-C dominant scales and externalizing problems and IIP-C submissive scales and internalizing problems. The pattern was more pronounced when the general distress factor was removed from the IIP-C. Social problems were uncorrelated with the IIP-C ipsatized scales.

**TABLE 3.** Correlation between IIP-C scales and YSR broadband scales and Social Problems

	PA = Domineering	BC = Vindictive	DE = Cold	FG = Socially avoidant	HI = Nonassertive	JK = Exploitable	LM = Overly nurturant	NO = Intrusive
Internalizing Problems	**0.287**	**0.368**	**0.48**	**0.539**	**0.52**	**0.464**	**0.623**	0.144
Externalizing Problems	**0.517**	**0.675**	**0.327**	0.15	0.053	0.072	**0.295**	**0.338**
Social Problems	**0.306**	**0.283**	**0.362**	**0.36**	0.238	0.097	0.2	0.002
Internalizing Problems	-0.205	--0.105	0.093	0.207	0.172	0.041	0.163	**-0.272**
Externalizing Problems	0.209	**0.455**	0.102	-0.195	**-0.324**	**-0.267**	-0.041	0.097
Social Problems	0.046	0.036	0.189	0.223	0.052	-0.143	-0.084	-0.23

1 *Note*. Bold = Significant correlation *p* > 0.05 (two tailed); Blue area = IIP-C scales based on ipsatized scores

## Discussion

### Summary of the study and results

The overall aim of the current study was to examine the psychometric properties of the IIP-C in a clinical sample of adolescents treated in specialty mental health services in Norway. This is the first study that examined the psychometric properties of the IIP-C used with a clinical adolescent population. Analyses included examining model fit, reliability of the scales, the factor structure, and validity by comparing it with a commonly used instrument in the child and adolescent mental health field—the Achenbach’s Youth Self Report.

The RMSEA did not confirm good model fit. However, the other three indices were more promising. The CFI, TLI and SRMR are incremental or approximate fit indexes, which means that they are estimates of increments from the baseline model to the hypothesized model. The literature suggests several cut-off guidelines. One frequently used guideline proposed by Hu & Bentler (42) recommends CFI and TLI close to .95 and SRMR below .80 as cut-off points for acceptable model fit. According to Marsh et al. (43), the guidelines proposed by Hu & Bentler (42) are too rigorous and stringent, which may lead to Type 1 error, that is erroneously rejecting the null hypothesis when it is true. Awang (44) proposed lower cut-off for CFI and TLI where >.90 means satisfactory fit. Given these criteria, the CFI and TLI from our study are above the .90 threshold. Further, the SRMR is well below the acceptable threshold of .80. While we agree that approximate fit indices are not substitutes and stringent statistical criteria increase our confidence in the IIP-C. Gurtman & Pincus (45) reasoned that aspiring for a geometrically sound circumplex structure may difficult. Further, what may be technically true may not necessarily mean it is practical and meaningful. Comparing our results with the Norwegian adult study (39) showed that our baseline CFA differed from theirs by .01 on the CFI and .02 on the TLI. However, we refrained from post-hoc fitting procedures to obtain acceptable fit indexes. Ours is a pilot study with a small sample size, and there are mixed recommendations in literature with a preference for theory-driven modifications over data-driven modifications (46).

Our goal is to use the IIP-C to assess adolescents’ interpersonal problems in a clinical setting, where there are currently no such measures. We, therefore, also conducted conventional reliability and validity analyses. The Cronbach’s alpha estimates of our study were .69 to .84, which was in the similar range reported by the clinical group in Norwegian adult study (39), which was .69 to .87 and the original research by Alden et al. (20), where the range was from .72 to .85. The lowest alpha value from our study was for PA, which indicates that the scale structure needs to be improved.

Examining the pattern of Cronbach’s alpha for the IIP-C scales, the highest alpha values were for the scales DE, FG, HI and JK and the lowest for scales NO and PA. This is a pattern we also noticed with other circumplex versions of the IIP for instance, the original study by Alden et al. (20), the Norwegian study by Monsen et al. (39) and the recent study by Bailey et al. (16). Similarly, the scales with the highest alpha value from our study were FG and HI (.82 and .84, which paralleled Alden’s data, where FG and HI also had the highest values (.85). These patterns show that our study is also susceptible to the same pattern of scale reliability as previous studies, and therefore not unusual.

According to the theory of circumplex structures, there should be high correlations between adjacent scales and negative correlations between scales directly opposite each other (47). The scales at orthogonal locations should be uncorrelated. The pattern of intercorrelations in our study showed the above pattern. Scales based on ipsatized scores showed a pattern of positive and negative correlations. By way of example, there was significant positive correlation between PA and BC, a significant negative correlation between PA and HI, and near-zero correlation between PA and its orthogonal subscale DE. Although not all correlations were statistically significant, the general tendency of intercorrelations from our study were in line with the theoretical assumptions of the interpersonal circumplex structures. Intercorrelation and PCA of the scales also showed the two dimensions of dominance and love, and the interpersonal circumplex structure emerged as seen in Figure 1. This eyeball measure shows we are on the right track.

Correlation between the IIP-C and YSR provided support for concurrent and discriminant validity. Thematically, the YSR internalizing scale captures withdrawal from social relationships and heightened experience of being lonely. The IIP-C scales on the submissive end of the circumplex capture lack of assertion, social inhibition and overly accommodating and compliant. As expected, we found a high correlation between, for instance, FG, HI and JK and internalizing problems. Likewise, the YSR externalizing scale that signifies behavioral and disruptive problems strongly correlated with the IIP-C dominant scales BC, PA and NO that reflect interpersonal problems with domineering, controlling and intrusion. These high correlations provide support for concurrent validity. Conversely, the internalizing and externalizing scales had low correlations with their theoretical opposites (see Table 3), thus providing support for discriminant validity. As noted earlier, the pattern was more pronounced when the general distress was removed from the IIP-C. Social problems had only a few low correlations with IIP-C, suggesting that the instruments may be measuring two different constructs.

The correlation between the LM (overly nurturant) and internalizing scale was unexpectedly high, even higher than the expected correlation between HI and internalizing problems. However, the same correlations based on ipsatized scales showed that HI had a higher correlation with internalizing problems than LM. This suggests that general distress played a role in the correlation between LM and internalizing problems. The content of LM captures the experience of doing too much for others at high personal cost to oneself. There is an implied sense of self-sacrifice and disappointment. Interestingly, this experience is more highly correlated with internalizing problems than the experience of being submissive, socially disconnected and inhibited. A longitudinal study showed that peer rejection in middle childhood is a pathway that puts adolescents on a trajectory to internalizing problems (48). Coupled with the growing importance of peer acceptance, it is possible that adolescents who feel they have invested a lot in the relationship and yet feel rejected may be equally, if not more, susceptible to internalizing problems.

## Limitations

The strengths of this study are its pioneer nature and clinical focus. Nevertheless, there are several limitations. The low sample size limited our ability to conduct additional analyses, for example, post-hoc model fitting and conduct split-sample analyses like EFA with a sub-sample and CFA with another sub-sample. The sample is also a specific clinical group primarily in treatment for internalizing problems, mainly depression. However, while this is a representative referral reason for adolescents in CAMHS Norway (49), a large sample representing a diversity of problem areas would contribute towards more robust analyses and applicability of the IIP-C. We envision future research to focus on two specific areas. First, is to explore if the developmental phase affects how adolescents respond to the IIP-C. Second, the advent of the internet, digital communication, and social media have dramatically influenced social life and interpersonal communication. Further, the year 2020 has seen the rise of the Covid-19 global pandemic and forced social isolation. It is unlikely that these events and trends have left interpersonal interactions and problems untouched. Future studies could explore how best to utilize the digital medium to assess interpersonal problems of adolescents, who are often first adopter of new technology and trends.
